# Heart Failure and Erectile Dysfunction: a Review of the Current Evidence and Clinical Implications

**DOI:** 10.1007/s11897-023-00632-y

**Published:** 2023-11-14

**Authors:** Maria Cristina Carella, Cinzia Forleo, Alessandro Stanca, Eugenio Carulli, Paolo Basile, Umberto Carbonara, Fabio Amati, Saima Mushtaq, Andrea Baggiano, Gianluca Pontone, Marco Matteo Ciccone, Andrea Igoren Guaricci

**Affiliations:** 1 Cardiovascular Disease Section, Interdisciplinary Department of Medicine, University of Bari Aldo Moro, Piazza Giulio Cesare 11, 70124 Bari, Italy; 2 Cardiology Unit, Madonna Delle Grazie Hospital, Matera, Italy; 3 Andrology and Kidney Transplantation Unit, Department of Emergency and Organ Transplantation-Urology, University of Bari Aldo Moro, Bari, Italy; 4 Department of Basic Medicine Neuroscience and Sense Organs, University of Bari Aldo Moro, Bari, Italy; 5 Perioperative Cardiology and Cardiovascular Imaging Department, Centro Cardiologico Monzino IRCCS, Milan, Italy

**Keywords:** Erectile dysfunction, Heart failure, Therapy, Sexual activity, Pathophysiology, Drug side effects

## Abstract

**Purpose of Review:**

Heart failure (HF) and erectile dysfunction (ED) are two common conditions that affect millions of men worldwide and impair their quality of life. ED is a frequent complication of HF, as well as a possible predictor of cardiovascular events and mortality. ED deserves more attention from clinicians and researchers.

**Recent Findings:**

The pathophysiology of ED in HF involves multiple factors, such as endothelial dysfunction, reduced cardiac output, neurohormonal activation, autonomic imbalance, oxidative stress, inflammation, and drug side effects. The diagnosis of ED in HF patients should be based on validated questionnaires or objective tests, as part of the routine cardiovascular risk assessment. The therapeutic management of ED in HF patients should be individualized and multidisciplinary, considering the patient’s preferences, expectations, comorbidities, and potential drug interactions. The first-line pharmacological treatment for ED in HF patients with mild to moderate symptoms (NYHA class I–II) is phosphodiesterase type 5 inhibitors (PDE5Is), which improve both sexual function and cardiopulmonary parameters. PDE5Is are contraindicated in patients who use nitrates or nitric oxide donors for angina relief, and these patients should be advised to avoid sexual activity or to use alternative treatments for ED. Non-pharmacological treatments for ED, such as psychotherapy or couples therapy, should also be considered if there are significant psychosocial factors affecting the patient’s sexual function or relationship.

**Summary:**

This review aims to summarize the most recent evidence regarding the prevalence of ED, the pathophysiology of this condition with an exhaustive analysis of factors involved in ED development in HF patients, a thorough discussion on diagnosis and management of ED in HF patients, providing practical recommendations for clinicians.

## Introduction

Heart failure (HF) is a chronic condition characterized by the inability of the heart to pump enough blood to meet the metabolic demands of the body [1]. HF affects more than 26 million people worldwide and is associated with high morbidity and mortality [2–4]. HF can cause various symptoms and complications that reduce the quality of life of patients and their partners, such as dyspnea, fatigue, edema, arrhythmias, depression, and sexual dysfunction [1].

Erectile dysfunction (ED) is defined as the persistent inability to achieve or maintain an erection sufficient for satisfactory sexual activity [5]. This condition affects more than 150 million men worldwide and its prevalence increases with age [5]. ED can have a negative impact on the psychological and emotional well-being of patients and their partners, as well as on their adherence to medical treatment [6]. Moreover, it can be also a marker of underlying cardiovascular disease (CVD), as it may precede the onset of HF symptoms by several years [7].

ED is a common complication of HF, affecting up to 81% of these patients [1]. The association between these two conditions is bidirectional and complex, involving multiple factors that impair the normal erectile function [7]. Moreover, the treatment of ED in HF patients poses several challenges and requires careful evaluation and individualization [1]. Therefore, it is important for clinicians to understand the causes, prevalence, diagnosis, and management of ED in HF patients, as well as the potential implications for their cardiovascular risk and prognosis.

This review aims to provide an updated overview of the current evidence and clinical implications regarding ED in HF patients.

## Prevalence of ED in HF

The prevalence of ED in HF patients is vastly increased, compared to the general population or patients with other cardiac diseases. ED is a common and distressing complication of HF that affects the quality of life and prognosis of these patients. It can be a marker of underlying cardiovascular disease and it can precede the onset of HF symptoms by several years [8].

The prevalence of ED in the general population is estimated to range from 2% in men younger than 40 years to 86% in men 80 years and older [9]. However, among HF patients, the prevalence of ED is much higher, with reports ranging between 74 and 84% [10–12].

The prevalence of ED in HF patients may vary according to several factors, such as the definition and measurement of ED, the severity and etiology of HF, the age and comorbidities of the patients, and the use of medications that may affect erectile function. However, it is clear that ED is a frequent and distressing complication of HF that affects the majority of these patients. Several studies have reported a correlation between the severity of HF and the severity of ED. For example, a study by Apostolo et al. found that none of the patients with peak oxygen consumption (VO2) < 10 mL/min/kg had normal or slightly impaired sexual function, while 34% of those with peak VO2 between 10 and 14 mL/min/kg had normal or slightly reduced sexual performance [13]. It revealed, also, a clear association between the New York Heart Association (NYHA) classification and both the prevalence and severity of ED, as measured by the International Index of Erectile Function (IIEF). In particular, the percentage of patients with normal or mildly impaired erectile function decreased from 70 to 50%, 10%, and almost 0% in patients with NYHA classifications of I, II, and IV, respectively [13]. Furthermore, within the same study, an in-depth analysis was conducted to evaluate the interaction between ED and other comorbidities. The results of this multivariate analysis revealed that, among the various comorbidities considered, only anemia and diabetes emerged as the comorbidities most frequently associated with ED [13]. Another study by Baumhäkel and Böhm found that patients with moderate or severe impairment of left ventricular ejection fraction (LVEF) had a significant increase of ED, thus identifying reduced LVEF as an independent risk factor for the development of ED in cardiovascular high-risk patients [14]. However, most of these studies have not showed adequate attention to the assessment of sexual function in patients with HF and preserved ejection fraction (HFpEF) and those with mid-range ejection fraction (HFmrEF), primarily focusing on patients with HF and reduced ejection fraction (HFrEF) [15]. This underscores the need for a comprehensive investigation into potential differences in sexual function among these distinct phenotypes within the HF spectrum. Such research could offer valuable insights into the relationship between LVEF and sexual function, shed light on alternative mechanisms, and potentially lead to different approaches in counseling and treatment [15].

All these findings suggest that the degree of impairment in cardiac function, exercise capacity, and the presence of comorbidities such as anemia and diabetes may influence the degree of impairment in erectile function. Therefore, assessing the severity of ED in HF patients may provide useful information about their cardiovascular risk, prognosis, and the potential impact of these comorbidities.

## Pathophysiology of ED in HF

The normal erectile function depends on a complex interaction between vascular, neural, hormonal, and psychological factors [5]. The main physiological mechanism involved in erection is the relaxation of the cavernosal smooth muscle cells (CSMCs), which allows the inflow of blood into the corpora cavernosa and the compression of the venous outflow [5]. This process is mediated by nitric oxide (NO), which is produced by endothelial cells (ECs) and neuronal cells (nNOS) in response to sexual stimulation. NO activates guanylate cyclase (GC) in CSMCs, which converts guanosine triphosphate (GTP) into cyclic guanosine monophosphate (cGMP) [5, 7]. cGMP then activates protein kinase G (PKG), which phosphorylates various proteins that regulate calcium homeostasis and contractility in CSMCs [5, 7]. The result is a decrease in intracellular calcium concentration and a relaxation of CSMCs. The cGMP is degraded by phosphodiesterase type 5 (PDE5), which is expressed in CSMCs and other tissues [5, 7]. The balance between NO production and cGMP degradation determines the duration and intensity of the erection.

**Fig. 1:**
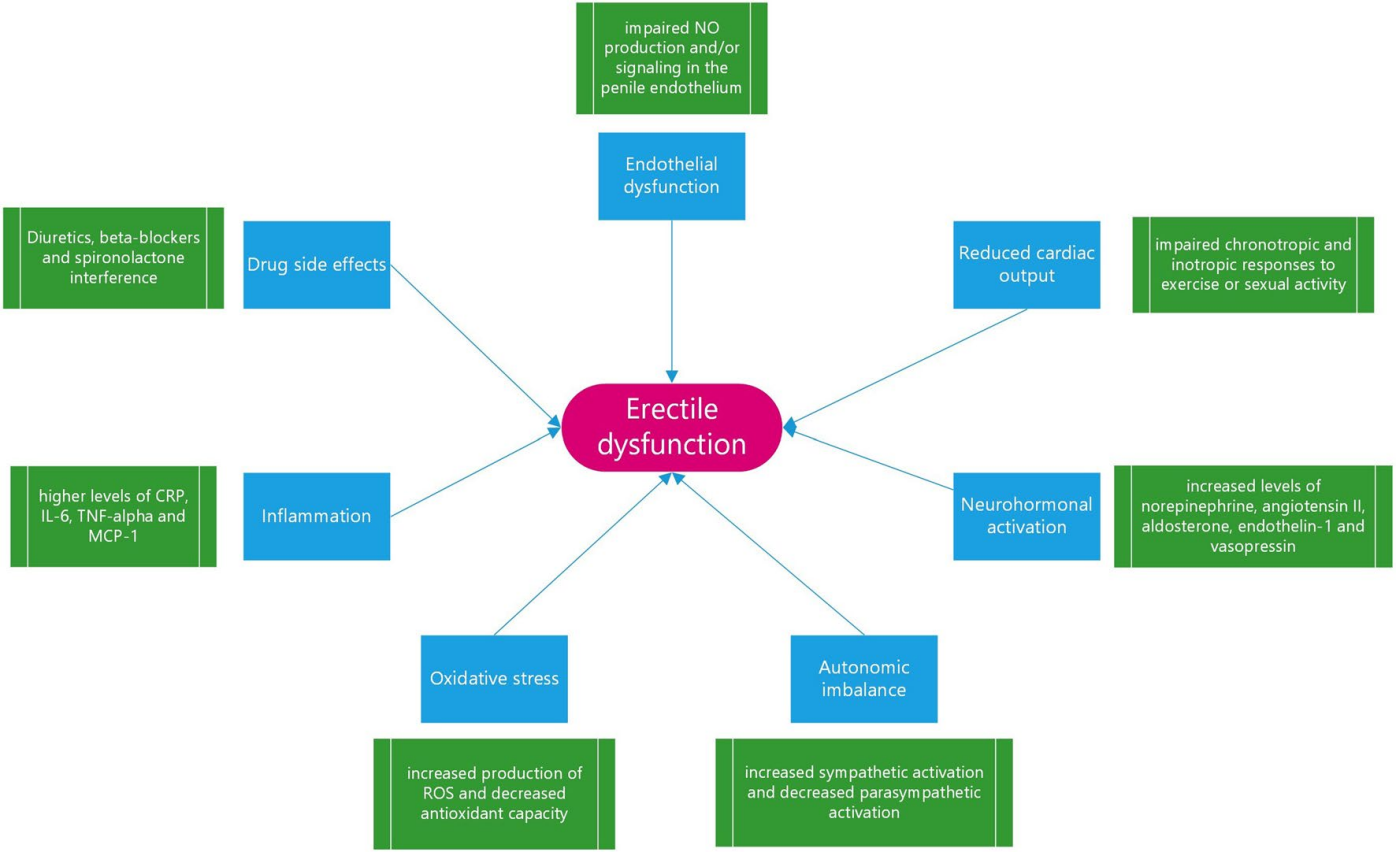
Organic factors involved in the development of erectile dysfunction in heart failure patients. NO, nitric oxide; CRP, C-reactive protein; IL-6, interleukin-6; TNF, tumor necrosis factor-alpha; MCP-1, monocyte chemoattractant protein-1; ROS, reactive oxygen species

The pathophysiology of ED in HF is complex and multifactorial, involving both organic and psychosocial factors [5, 16]. The main organic factors are endothelial dysfunction, reduced cardiac output, neurohormonal activation, autonomic imbalance, oxidative stress, inflammation, and drug side effects [5, 16]. The factors involved are summarized in Fig. 1.

The physiopathology of erectile dysfunction in heart failure patients is complex and multifactorial. Several organic factors may lead to its development. A detailed description of each factor is provided in the text.

### Endothelial Dysfunction

Endothelial dysfunction is a key mechanism that impairs the vasodilation of the penile arteries and the relaxation of the CSMCs, leading to impaired erectile function [16]. It is characterized by a reduced bioavailability of NO, due to decreased synthesis, increased degradation, or impaired signaling [16]. Endothelial dysfunction can be caused by various factors, such as aging, hypertension, diabetes, dyslipidemia, smoking, obesity, inflammation, and oxidative stress [17, 18]. Endothelial dysfunction is also a hallmark of HF, as it contributes to the progression of atherosclerosis, the development of cardiac remodeling, and the activation of neurohormonal systems [17, 18]. Therefore, HF patients have a double burden of endothelial dysfunction, affecting both the systemic and the penile circulation [17, 18].

Several studies have shown that HF patients have reduced endothelium-dependent vasodilation in the brachial artery and in the cavernosal artery, compared to healthy controls or patients with coronary artery disease [17]. Moreover, HF patients have lower levels of circulating NO metabolites and higher levels of asymmetric dimethylarginine (ADMA), an endogenous inhibitor of NO synthase (NOS) [19]. These findings indicate that HF patients have impaired NO production and/or signaling in the penile endothelium, which may explain their reduced erectile function [19].

### Reduced Cardiac Output

Reduced cardiac output is another factor that can affect the blood flow to the penis and the oxygen delivery to the erectile tissue. HF patients have lower resting and peak cardiac output than healthy controls [20]. Moreover, HF patients have impaired chronotropic and inotropic responses to exercise or sexual activity [21], which limit their ability to increase their cardiac output when needed. Therefore, HF patients may have insufficient blood flow and oxygen supply to the penis during sexual stimulation, resulting in impaired erection.

Several studies have shown that HF patients have lower penile blood flow at baseline and during pharmacological or visual stimulation than healthy controls [22, 23]. HF patients have impaired penile hemodynamics and oxygenation, leading to ED.

### Neurohormonal Activation

Neurohormonal activation controls vascular and neural pathways involved in erectile function. HF patients have increased levels of several neurohormones, such as norepinephrine, angiotensin II, aldosterone, endothelin-1, and vasopressin [24]. These neurohormones can have deleterious effects on the cardiovascular system, such as vasoconstriction, sodium retention, cardiac hypertrophy, and fibrosis. Moreover, they can also interfere with the erectile function by reducing NO bioavailability, increasing oxidative stress and inflammation, impairing endothelial function and smooth muscle relaxation, altering autonomic balance, and modulating central nervous system activity [25].

Higher plasma levels of norepinephrine, angiotensin II, aldosterone, and endothelin-1 were detected in HF patient as compared to healthy controls [26].

### Autonomic Imbalance

Autonomic imbalance can affect the neural pathways involved in erectile function. The normal erectile function depends on a balance between sympathetic and parasympathetic nervous system activity [27]. The parasympathetic nervous system mediates the initiation of erection by releasing NO from nNOS in response to sexual stimulation. The sympathetic nervous system mediates the maintenance of erection by releasing norepinephrine from adrenergic nerves, which stimulates alpha-adrenergic receptors on CSMCs and causes their contraction. The sympathetic nervous system also mediates the ejaculation and detumescence by releasing norepinephrine from adrenergic nerves, which stimulates alpha-adrenergic receptors on the bulbocavernosus and ischiocavernosus muscles and causes their contraction [28]. Autonomic imbalance can alter the balance between sympathetic and parasympathetic nervous system activity, which is essential for initiating and maintaining an erection [29].

HF patients have increased sympathetic activation and decreased parasympathetic activation, resulting in autonomic imbalance [30]. Moreover, they have higher plasma levels of norepinephrine and lower levels of heart rate variability than healthy controls [31]. These findings indicate that HF patients have increased sympathetic tone and reduced parasympathetic tone, inducing ED.

### Oxidative Stress

Oxidative stress is a state of imbalance between the production of reactive oxygen species (ROS) and the antioxidant defense mechanisms. ROS are molecules that contain oxygen and have high reactivity, such as superoxide anion (O2–), hydrogen peroxide (H2O2), and hydroxyl radical (OH–). ROS can cause oxidative damage to various biomolecules, such as lipids, proteins, and DNA, impairing their structure and function [32, 33]. ROS can also modulate the activity of various enzymes and signaling molecules, such as NOS, GC, PKG, and PDE5, affecting their regulation of erectile function [34].

HF patients have increased oxidative stress, due to increased production of ROS and decreased antioxidant capacity [35]. Higher levels of oxidative stress markers, such as malondialdehyde (MDA), 8-hydroxy-2′-deoxyguanosine (8-OHdG), and nitrotyrosine (NT), were observed in these patients as compared healthy controls [36]. To summarize, HF patients have increased oxidative damage and reduced antioxidant defense, which may impair their erectile function.

### Inflammation

Inflammation plays a central role in ED. It represents a complex biological response to tissue injury or infection, involving various cells, mediators, and pathways. Inflammation can cause vasodilation, increased vascular permeability, leukocyte infiltration, cytokine release, and tissue damage [37, 38]. In addition, it can also modulate the activity of various enzymes and signaling molecules, such as NOS, GC, PKG, and PDE5, affecting their regulation of erectile function [39]. HF patients have increased inflammation, due to a marked activation of immune cells and cytokines, with higher levels of inflammatory markers, such as C-reactive protein (CRP), interleukin-6 (IL-6), tumor necrosis factor-alpha (TNF-alpha), and monocyte chemoattractant protein-1 (MCP-1), than healthy controls [40]. HF patients have increased inflammatory response and tissue damage, which may promote ED.

### Drug Side Effects

Several drugs recommended in the treatment of HF can interfere with the hormonal, vascular, and neural pathways involved in erectile function. Therefore, these drugs should be used at the lowest effective doses and monitored for their effects on sexual function. If possible, alternative drugs with less or no impact on sexual function should be considered.

The main classes of drugs that can induce ED in HF patients are diuretics, beta-blockers, and spironolactone [25] (Table 1). Diuretics are drugs that increase the excretion of water and electrolytes by the kidneys, reducing the blood volume and the preload of the heart. These drugs are essential for the treatment of HF patients with fluid retention and edema [41]. However, diuretics can also affect the erectile function by causing hypovolemia, hypotension, electrolyte imbalance, dehydration, and activation of the renin–angiotensin–aldosterone system (RAAS) [42]. They can reduce the production of testosterone and increase the production of prolactin, affecting the hormonal regulation of sexual function [43]. The main types of diuretics involved are thiazide and loop diuretics. Beta-blockers are drugs that block the beta-adrenergic receptors on various tissues, reducing the effects of sympathetic stimulation. These drugs are essential for the treatment of patients with HFrEF (class I recommendation according to the latest European guidelines [44]), as they reduce the heart rate, blood pressure, myocardial oxygen consumption, and cardiac remodeling. However, beta-blockers may induce ED by reducing the cardiac output, blood flow, and oxygen delivery to the penis [45]. Beta-blockers can also impair the neural regulation of sexual function by blocking the beta-adrenergic receptors on the penile smooth muscle and nerves [46]. The main types of beta-blockers that can affect erectile function are nonselective beta-blockers and beta-1 selective blockers. Spironolactone is a mineralocorticoid receptor antagonist acting on various tissues, reducing the effects of aldosterone. It shares the same remodeling effects as beta-blockers [47]. Relevant side effects are hyperkalemia, gynecomastia, impotence, and decreased libido [48]. Spironolactone can also interfere with the hormonal regulation of sexual function by inhibiting the synthesis of testosterone and enhancing the conversion of testosterone to estradiol [49].

**Table 1:** Drugs used in the treatment of heart failure causing erectile dysfunction as side effect

Drug class	Examples	Mechanisms of side effects
Diuretics	Loop diuretics - Furosemide - Bumetanide - Torasemide - Ethacrynic acid Thiazide diuretics - Hydrochlorothiazide - Chlorthalidone - Indapamide - Metolazone	- Hypovolemia and hypotension - Electrolyte imbalance - Dehydration and activation of the renin–angiotensin–aldosterone system - Reduced production of testosterone and increased production of prolactin
Beta-blockers	Nonselective beta-blockers - Propranolol - Nadolol - Timolol - Sotalol Beta-1 selective blockers - Atenolol - Bisoprolol - Metoprolol	- Reduced cardiac output, blood flow, and oxygen delivery to the penis - Blocked beta-adrenergic receptors on the penile smooth muscle and nerves
Mineral receptor antagonist	Spironolactone	- Gynecomastia - Impotence and decreased libido - Inhibited synthesis of testosterone and enhanced conversion of testosterone to estradiol

Several drugs recommended in the therapeutic management of heart failure may cause erectile dysfunction. The development of this side effect is related to several mechanisms. When prescribing these drugs, erectile dysfunction should be prompt recognized as side effect and the molecules implicated should be down titrated at the lowest effective doses or replaced with a molecule of the same class having a less or no impact on sexual function.

## Diagnosis of ED in HF

**Fig. 2:**
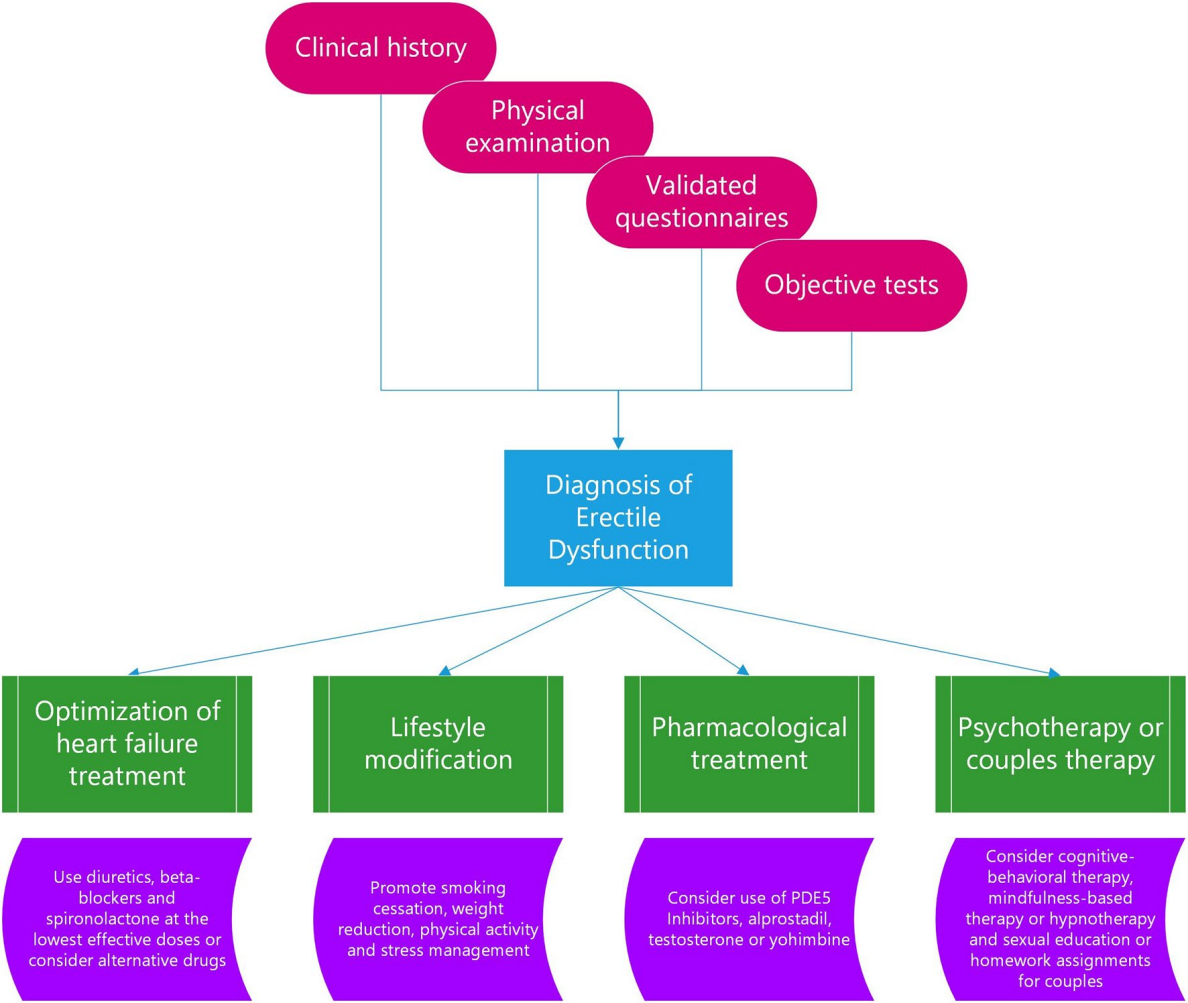
Management of erectile dysfunction in heart failure patients. PDE5, phosphodiesterase type 5

The diagnosis of ED in HF patients is a crucial step to identify and treat this common and distressing condition, as well as to assess the cardiovascular risk and prognosis of these patients. The diagnosis should be based on a comprehensive and multidisciplinary approach, involving clinical history, physical examination, validated questionnaires, and objective tests as shown in Fig. 2 [16].

The clinical history represents the first and most important step in the diagnosis of ED in HF patients. It should include questions about the onset, duration, frequency, severity, and causes of ED, as well as its impact on the patient’s quality of life, relationship, and sexual satisfaction. It should also include questions about the patient’s sexual history, preferences, expectations, and goals, as well as any psychosocial factors that may affect his sexual function or motivation. The patient’s medical history should be investigated, especially regarding any comorbidities or medications that may cause or contribute to ED [50]. The clinical history should be conducted in a respectful and empathic manner, using clear and non-judgmental language.

The physical examination is the second step in the diagnostic evaluation. It should include a general examination to assess the patient’s vital signs, body mass index (BMI), cardiovascular status, and signs of low testosterone (such as gynecomastia, reduced body hair, or muscle mass) [51].

Validated questionnaires are useful tools to quantify the degree and severity of ED, as well as to monitor its response to treatment. The most widely used questionnaire for ED is the *International Index of Erectile Function-5 (IIEF-5)*, which consists of 15 questions that assess the patient’s erectile function during the past 4 weeks [52]. The IIEF-5 score ranges from 5 to 25, with lower scores indicating more severe ED. The IIEF-5 score can be classified into five categories: no ED (22–25), mild ED (17–21), mild to moderate ED (12–16), moderate ED (8–11), and severe ED (5–7) [53]. The IIEF-5 questionnaire has been validated in various languages and populations, including HF patients [54].

Objective tests are the last step in the diagnosis of ED in HF patients. They are used to confirm the diagnosis of ED, to identify its etiology, and to exclude any organic causes. The most widely used objective tests for ED are:

- Nocturnal penile tumescence (NPT) test: it measures the frequency and duration of spontaneous erections during sleep using a device attached to the penis. A normal NPT test indicates that there is no organic cause for ED and that it is mainly psychogenic. An abnormal NPT test indicates that there is an organic cause for ED and that it may be vascular or neurogenic [55].
- Penile Doppler ultrasound: the blood flow within the penile arteries before and after an intracavernosal injection of a vasoactive agent (such as prostaglandin E1 or papaverine) was assessed with this technique. A normal penile Doppler ultrasound shows an increase in peak systolic velocity (> 30 cm/s) and a decrease in end-diastolic velocity (< 5 cm/s) after injection, indicating adequate arterial inflow and venous outflow. An abnormal penile Doppler ultrasound shows a decrease or no change in peak systolic velocity (< 30 cm/s) or an increase or no change in end-diastolic velocity (> 5 cm/s) after injection, indicating arterial insufficiency or venous leakage [56–59].
- Penile dynamic infusion cavernosometry and cavernosography: these tests measure the intracavernosal pressure and volume before and after an intracavernosal injection of a vasoactive agent (such as prostaglandin E1 or papaverine), while simultaneously injecting saline into the corpora cavernosa under fluoroscopic guidance. A normal penile dynamic infusion cavernosometry shows a rapid increase in intracavernosal pressure after injection, indicating adequate arterial inflow. A normal penile dynamic infusion cavernosography shows a smooth and symmetrical filling of the corpora cavernosa without any leakage or extravasation of contrast, indicating adequate venous outflow. An abnormal penile dynamic infusion cavernosometry shows a slow or no increase in intracavernosal pressure after injection, indicating arterial insufficiency. An abnormal penile dynamic infusion cavernosography shows an irregular or asymmetrical filling of the corpora cavernosa with leakage or extravasation of contrast, indicating venous leakage [60–62].
- Penile biothesiometry: this test measures the vibratory threshold of the penile skin using a biothesiometer, which is a device that delivers variable frequencies of vibration to the glans and shaft of the penis. It is based on the principle that the threshold of vibration detection is related to the integrity of the nerve fibers that innervate the penis. Penile biothesiometry can be used to diagnose penile neuropathy, which is a common cause of ED, especially in patients with diabetes, Peyronie’s disease, or spinal cord injury. Penile biothesiometry can also be used to assess changes in penile sensitivity after penile reconstructive procedures that may compromise penile sensation [63]. A novel parameter that can be derived from penile biothesiometry is the penile sensitivity ratio (PSR), which is calculated by dividing the vibratory threshold of the penile glans or shaft by the vibratory threshold of the index finger or thigh. The PSR is inversely correlated with penile sensitivity, meaning that a higher PSR indicates a lower sensitivity. The PSR can help identify patients with diminished penile sensitivity and evaluate the association between penile sensitivity and various factors such as age, diabetes, ejaculatory dysfunction, and Peyronie’s disease [64].

The choice of objective tests for ED in HF patients depends on the local availability of the equipment, the expertise of the operator, the preference of the patient, and the suspected etiology of ED. In general, penile Doppler ultrasound is the most widely used and recommended objective test for ED in HF patients, as it is noninvasive, reliable, reproducible, and informative [58]. Penile dynamic infusion cavernosometry and cavernosography are more invasive, expensive, and time-consuming tests that are reserved for patients with suspected venous leakage who are candidates for surgical treatment [65]. Penile biothesiometry is a simple and inexpensive test that can be used as an adjunct to other tests to assess the neurogenic component of ED [66].

## Therapeutic Management of ED in HF

The therapeutic management of ED in HF patients should be individualized and multidisciplinary, taking into account the patient’s preferences, expectations, comorbidities, and potential drug interactions. The main goals are the improvement of the sexual function and quality of life of the patient and his partner, the optimization the HF treatment, and the modification of any lifestyle or behavioral factors that may contribute to ED. The management of ED in HF patients involves four areas: optimization of HF treatment, lifestyle modification, pharmacological treatment with PDE5Is or other agents, and non-pharmacological treatment with psychotherapy or couples therapy. This approach is summarized in Fig. 2.

### Optimization of HF Treatment and Lifestyle Modification

The optimization of HF treatment and any lifestyle or behavioral changes play a key role in the therapeutic approach. Beneficial effects are evident not only on the erectile function but also on the cardiovascular performance and prognosis of the patient [67]. Optimization of HF treatment involves adjusting the doses and combinations of drugs according to the current guidelines and the patient’s clinical status. Some drugs used to treat HF, such as diuretics, beta-blockers, and spironolactone, can interfere with the hormonal, vascular, and neural pathways involved in erectile function [51]. Therefore, these drugs should be used at the lowest effective doses and monitored for their effects on sexual function. If possible, alternative drugs with less or no impact on sexual function should be considered, such as angiotensin-converting enzyme inhibitors (ACEIs), angiotensin receptor blockers (ARBs), nitrates, or nebivolol [68–70].

Lifestyle modification is based upon the removal of any modifiable risk factors for ED and CVD, such as smoking habit, obesity, sedentary behavior, and stressful lifestyle. Smoking habit is a major risk factor for ED and CVD, as it causes endothelial dysfunction, oxidative stress, and inflammation [71]. Smoking cessation can improve both erectile function and cardiovascular function in HF patients [72]. Weight loss may induce beneficial effects on both erectile and cardiovascular function in obese HF patients by reducing insulin resistance, inflammation, and oxidative stress [73]. Physical activity can improve both erectile function and cardiovascular function in HF patients by enhancing endothelial function, increasing NO bioavailability, reducing oxidative stress and inflammation, improving autonomic balance, and modulating central nervous system activity [74, 75]. Stress management may have a positive role in ED, reducing sympathetic activation, cortisol levels, oxidative stress, and inflammation [76, 77].

### Pharmacological Treatment With PDE5Is or Other Agents

The second area in the management of ED in HF patients is the prescription of a pharmacological treatment with PDE5Is or other agents. This approach improves the sexual function and quality of life of the patient and his partner. PDE5Is are the first-line pharmacological treatment for ED in HF patients with mild to moderate symptoms (NYHA class I–II) [78, 79]. PDE5Is enhance the effect of NO on the penile vasculature and smooth muscle, improving erectile function [80]. Several studies have shown that PDE5Is are safe and effective in HF patients with mild to moderate symptoms (NYHA class I–II), as they improve not only their sexual function but also their cardiopulmonary parameters and quality of life [79]. PDE5Is have also beneficial effects on endothelial function, NO bioavailability, oxidative stress, inflammation, and cardiac remodeling in animal models of HF [81]. However, PDE5Is are contraindicated in patients who use nitrates or nitric oxide donors for angina relief, as they can cause severe hypotension and potentially fatal outcomes [82]. Therefore, these patients should be advised to avoid sexual activity or to use alternative treatments for ED.

Other pharmacological agents that can be used for ED in HF patients are alprostadil, testosterone, or yohimbine. Alprostadil is a synthetic prostaglandin E1 that induces vasodilation and smooth muscle relaxation in the penis. It can be administered as an intracavernosal injection or an intraurethral pellet [83]. Alprostadil is effective for ED regardless of its etiology, but it has some drawbacks such as pain at the injection site or urethral irritation [84]. Testosterone is the main male sex hormone that regulates sexual function and libido. It can be administered as an intramuscular injection, a transdermal patch, or a gel [85]. Testosterone can improve erectile function and sexual desire in hypogonadal HF patients, but it has some risks such as prostate enlargement, polycythemia, and cardiovascular events [86]. Yohimbine is an alpha-2 adrenergic receptor antagonist that enhances the release of norepinephrine from adrenergic nerves, stimulating erectile function. It can be administered as an oral tablet [87], improving erectile function and sexual satisfaction in HF patients, but it has some side effects such as anxiety, hypertension, and tachycardia [88].

### Non-pharmacological Treatment With Psychotherapy or Couples Therapy

Non-pharmacological treatment with psychotherapy or couples therapy has an important role, especially if there are significant psychosocial factors affecting the patient’s sexual function or relationship. Psychotherapy is a form of psychological intervention that aims to help the patient cope with his emotional and cognitive aspects of ED, such as anxiety, depression, low self-esteem, guilt, or shame. Main goals are the identification and correction of any negative thoughts or beliefs that may interfere with his sexual function or satisfaction [6]. Psychotherapy can be delivered individually or in group sessions, using various techniques such as cognitive-behavioral therapy (CBT), mindfulness-based therapy, or hypnotherapy [89–91]. Couples therapy is a form of psychological intervention that aims to support the patient and his partner communicate better about their sexual concerns and expectations, enhance their intimacy and trust, and resolve any conflicts or issues that may affect their sexual function or satisfaction. It can also help the partner cope with his or her own emotional reactions to the patient’s ED, such as frustration, anger, resentment, or rejection [92]. Couples therapy can be delivered in conjoint or separate sessions, using various techniques such as sensate focus exercises, sexual education, or homework assignments [93].

### Indications for Sexual Activity in HF

The indications for sexual activity in HF patients depend on the severity of their symptoms, their functional capacity, and their cardiovascular risk. Sexual activity can be considered as a form of physical exercise that imposes a certain degree of cardiac stress and oxygen demand. Therefore, it can be safe for HF patients who have mild to moderate symptoms (NYHA class I–II), a good functional capacity, and a low cardiovascular risk (no history of angina, arrhythmias, syncope, or HF exacerbation) [94, 95]. On the other hand, sexual activity can be risky for HF patients with severe symptoms (NYHA class III–IV), a poor functional capacity, and a high cardiovascular risk [96].

HF patients who are not eligible for sexual activity should be counseled about the risks and benefits of sexual activity, the alternative treatments for ED, and the non-coital forms of sexual expression [97]. They should also be referred to a cardiologist for further evaluation and optimization of their HF treatment.

The figure summarizes the management of erectile dysfunction in heart failure patients. The diagnostic algorithm is based on clinical history and physical examination. Subsequently, validated questionnaires are required to confirm the diagnosis. A more objective evaluation of erectile performance may be required in selected cases with specific tests and techniques. The therapeutic management is multidisciplinary and should involve four areas to obtain an improvement on the sexual function and the quality of life of patients. Beneficial effects may be also observed on the cardiovascular performance and prognosis.

## Conclusions

ED is a common and distressing complication of HF that deserves more attention from clinicians and researchers. This condition can affect the quality of life and prognosis of HF patients and can also be a sign of underlying CVD. Therefore, screening for ED should be part of the routine evaluation of HF patients and its management should be individualized and multidisciplinary. PDE5Is are the first-line pharmacological treatment in HF patients with mild to moderate symptoms (NYHA class I–II), as they improve their sexual function, cardiopulmonary parameters, and quality of life. However, PDE5Is are contraindicated in patients treated with nitrates or nitric oxide donors for angina relief, and these patients should be advised to avoid sexual activity or to use alternative treatments for ED. Non-pharmacological treatments for ED, such as psychotherapy or couples therapy, should also be considered if there are significant psychosocial factors affecting the patient’s sexual function or relationship.

## Data Availability

No new data were created for this review.
